# Neuraminidase activity in pediatric nephrotic syndrome

**DOI:** 10.1007/s00467-026-07163-3

**Published:** 2026-01-17

**Authors:** Xhuliana Kajana, Agnese Spennacchio, Andrea Angeletti

**Affiliations:** Nephrology, Dialysis and Transplantation Unit, IRCCS Istituto Giannina Gaslini, Genoa, Italy

**Keywords:** Nephrotic syndrome, Neuroaminidase, Sialidase, Sialic acid, Proteinuria

Nephrotic syndrome (NS) is characterized by increased glomerular permeability resulting from podocyte injury and disruption of the glomerular filtration barrier. Although its pathogenesis is thought to involve immune-mediated mechanisms, the molecular events underlying disease onset and relapse, often triggered by infectious episodes, remain incompletely understood [1]. Sialic acids are terminal monosaccharides and fundamental components of glycoproteins and glycolipids, where they regulate molecular interactions. Sialic acids are also key constituents of the polyanionic surface of podocytes and the glomerular basement membrane, contributing to structural stability [2]. Previous experimental and human studies have shown that glomerular hyposialylation may be associated with podocyte foot process effacement and proteinuria [3, 4]. Of note, in steroid-dependent NS, reduced IgM sialylation has been linked to increased T-cell–mediated podocyte injury and reduced steroid responsiveness [5]. Therefore, although the loss of sialic acid is recognized as having a possible pathogenic role in NS, the mechanisms by which altered sialylation may modulate disease activity remain incompletely defined.

In this context, a well-recognized clinical feature of pediatric NS is the frequent temporal association between disease onset or relapse and infectious episodes. Several pathogens express neuraminidase (sialidase), an enzyme that facilitates pathogen dissemination by cleaving sialic acid residues from host glycoproteins. Therefore, we hypothesized that neuraminidase activity may be associated with clinical disease activity in NS.

**Fig. 1:**
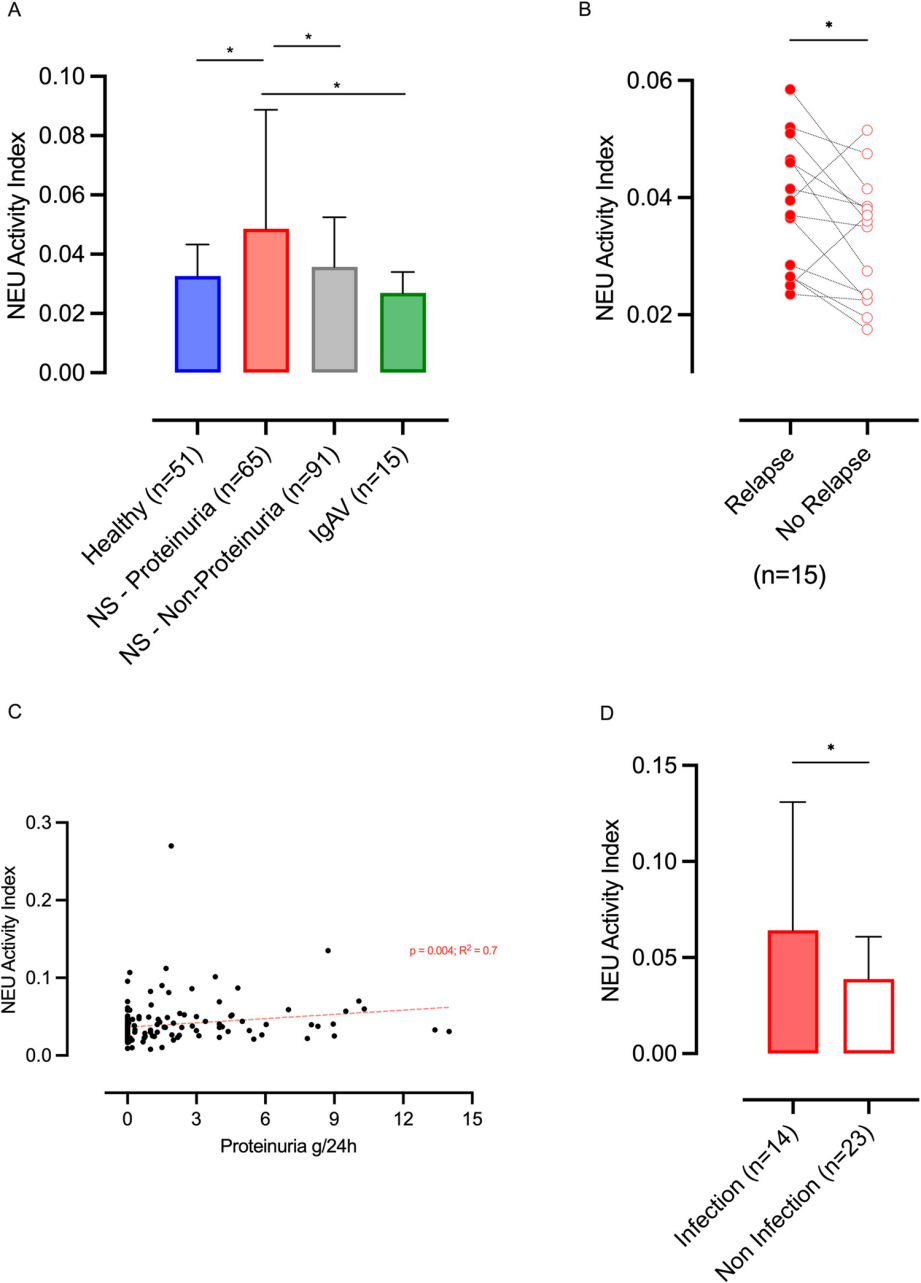
Serum neuraminidase activity in pediatric nephrotic syndrome and its association with disease activity. **A** Serum neuraminidase activity index in healthy controls (*n* = 51), patients with nephrotic syndrome (NS) during active proteinuric phases (*n* = 65), patients with NS in non-proteinuric phases (*n* = 91), and disease controls with IgA vasculitis–associated nephritis (IgAV, *n* = 15). **B** Paired intra-individual comparison of serum neuraminidase activity in 15 patients studied both during relapse (nephrotic proteinuria) and remission. Each pair of connected dots represents measurements obtained from the same patient. **C** Correlation between serum neuraminidase activity index and degree of proteinuria (g/24 h) in patients with NS. Linear regression analysis, *p* = 0.004, *R*^2^ = 0.7. **D** Serum neuraminidase activity in patients with NS experiencing relapse, stratified according to the presence (*n* = 14) or absence (*n* = 23) of a documented infectious episode within the month preceding relapse, as assessed by review of hospital clinical records. Data are shown as mean ± SD; *p < 0.05

To explore this hypothesis, we measured serum neuraminidase activity in pediatric patients with NS and in matched controls. The total serum neuraminidase activity, without isoform specificity, was measured by a fluorometric enzymatic assay (Amplex™ Red Neuraminidase Assay Kit, Thermo Fisher Scientific). Samples were measured in duplicate. Overall, 156 children (9.8 ± 6.3 years old) with a history of NS and 64 controls (healthy children and disease controls with IgA Nephritis) were tested. As the main findings, serum neuraminidase activity was significantly increased in patients with NS during active proteinuric phases compared with all control groups (Fig. 1A), supporting a specific association with disease activity, independent of prior disease course or ongoing therapy.

To further strengthen this observation, we performed paired analyses in 15 patients both during relapse and remission. The intra-individual comparisons demonstrated consistently higher serum neuraminidase activity during active disease compared with remission (Fig. 1B). Importantly, serum neuraminidase activity showed a significant positive correlation with the degree of proteinuria (Fig. 1C).

We next investigated the relationship between neuraminidase activity and infectious triggers. Among patients experiencing relapse, review of clinical records identified infectious episodes within the preceding month. Serum neuraminidase activity was significantly higher in patients with recent infection compared with those without documented infectious episodes (Fig. 1D). Reported infections mainly involved the upper respiratory tract, consistent with pathogens that may express neuraminidase; however, detailed microbiological or inflammatory patterns were not systematically available.

In conclusion, we demonstrate that serum neuraminidase activity is increased in active pediatric NS and closely associated with proteinuria and infection-related relapses. These findings reinforce previous observations and highlight previously underexplored mechanistic aspects of NS. Specifically, it remains to be determined whether neuraminidase activity exerts direct effects on podocytes or primarily targets immune components, as previously reported, and whether desialylation is reversible and temporally linked to specific disease phases, potentially informing targeted therapeutic strategies, including approaches aimed at restoring sialylation.
